# The Importance of Diagnosis and Treatment in Diseases of the Stomach

**Published:** 1902-08

**Authors:** Jno. C. McAfee

**Affiliations:** Macon, Ga.; Member Medical Association of Georgia and American Medical Association


### THE IMPORTANCE OF DIAGNOSIS AND TREATMENT IN DISEASES OF THE STOMACH.*

BY JNO. C. McAFEE, A.B., M.D., MACON, GA.

Member Medical Association of Georgia and American Medical Association.

In the consideration of any phase of our professional work, or any single division or subject thereof, it is not only our pride and satisfaction, but also our immense benefit, and that of our patient, when our services are required in any given case, first of all to discover what it is we have to deal with. No sane, conscientious surgeon would attempt to operate upon a patient, except for two reasons, which are clear and unmistakable, viz.: First, to relieve his patient, or prolong life; second, to discover the cause of the illness, in order that it may be removed. In these progressive

*Read before the Macon Medical Society.

days of medicine and surgery, when the abdominal cavity, and even the cranium itself, together with the cavity of the spinal column, are invaded for the sake of diagnosis, and tuberculin is injected into patients for diagnostic purposes; when the Roentgen rays are brought into play with their immense and increasing value in diagnosis, especially in surgery, and bacteriologists are diligently searching for pathogenic germs yet undiscovered; in these times when the energy of the profession is focused upon diagnosis, it is a strange and unexplainable fact that the large field of digestive troubles is almost completely pushed aside, especially by the physicians of the South. How many of us have text-books on diseases of the stomach and intestines in our libraries?

The fact that diagnosis in any case is first in importance is one that none of us would care to gainsay, as we know that our hypothetical position would be untenable. There can be no question in the mind of any progressive, thinking physician that the field of empiricism, or experimentation, in medicine, is day by day passing away, and its place being supplied by a scientific exactness and certainty in exploring the pathologic conditions of our patients that is encouraging and comforting to contemplate, and our patients are reaping the benefit in their personal betterment. No man can question the fact that the treatment of any case whatever, in medicine or surgery, is purely empirical until the diagnosis of the case is made, and the attending physician knows the nature of the ailment he is called upon to treat.

Turning our attention to the field of diseases of the stomach, or digestive troubles, it is discouraging, indeed, to note the indifference of the profession to, and lack of interest in, the diagnosis and treatment of the ills that this class of sufferers is heir to. Is this a sin of the general practitioner? Not at all, for digestive diseases, or diseases of the stomach and intestines, constitute a specialty second in importance to none, for the well-being of the entire physical, and also frequently mental, economy is probably more directly influenced by derangements of the digestive tract than by derangement of any other portion of the body.

Nothing can be more important to the body than nutrition, and when this is disturbed, the body passes into decay just in proportion to the disturbance. These facts are axiomatic, and none but the ignorant or prejudiced will essay to deny them.

Who of us will deny that the step of first importance in the treatment of tuberculosis is the improvement of nutrition and proper feeding? This we know to be our sheet-anchor in the treatment of this the most widely fatal of all diseases; and if this be true—as we believe it is—and if cases of tuberculosis actually recover and get well under this treatment principally—as we believe they do—then who can successfully maintain that the patient would ever have contracted the disease had his digestion and assimilation been normal and healthy? And if so dread a disease as tuberculosis can be kept off by normal digestion and assimilation, why may not this be true with less powerful and dangerous diseases? I believe it is so. Reasoning logically then, the best method of prophylaxis against disease, certainly some of the worst types, is a normal, healthy digestive system, and if this does not amount to proof against diseases, it is certainly the strongest foundation upon which to base any treatment of any disease.

It is only within the last decade or two that very much information of value has been at our disposal regarding the diagnosis and treatment of diseases of the stomach, and for this reason is not much disseminated.

Pioneer workers in this field of pathology, such as Kussmaul, Penzoldt, Enold, Nothnagel, and later Hemmeter, Einhorn, Turck and Salzer in this country, together with a host of others, have given us sufficient information on the subject of digestive pathology to enable us to successfully cope with those diseases of the stomach and intestines now recognized as curable. "Little over a decade ago it was customery to designate all stomach diseases that were not acute, and that could not be diagnosed as ulcer, dilatation, or carcinoma, as 'chronic gastric catarrh'". With modern methods of diagnosis, however, the word "catarrh" is practically discarded, as it signifies a superficial inflammation, whereas we know now that the chronic inflammations of the stomach with which we have to deal involve all the coats of this glandular secreting organ, "and that nearly always one or another of these coats suffers most."

Also, "by these methods of analysis the gastric neuroses have been recognized as separate and distinct," and it is known to be a fact that the patient not only suffers more from the neuroses than from most of the organic lesions, as a rule, but that these neuroses affect gastric secretion, and, if left uncorrected, will continue, and eventually lead to permanent organic changes. And, of course, the sooner beginning changes of structure are intercepted and corrected, the less likely is the patient to suffer permanent pathological degeneration of his stomach tissues.

Now, speaking comprehensively in regard to treatment, as it is not the aim of this paper to discuss treatment, and my time will not permit me to discuss it now, we may say that, with the exception of malignancy, there is no form of stomach trouble that modern treatment cannot cure or benefit. Most stomach troubles are amenable to cure, and I have known the severest type of atrophic gastritis, in which the digestive power of the stomach was completely gone, and in which anorexia was absolute, to be so entirely relieved by treatment that the patient was not aware that he had a stomach. The plans of treatment, which I mean to discuss at another time, are simple, and also refreshing and agreeable to the patient, but require a good deal of care and time on the part of the physician, as most of the treatment has to be carried out in the office. With our present knowledge of this subject, there is no longer any use or necessity in temporizing or experimenting with these sufferers, or even offering them nothing at all in the way of relief, for the reason that the case cannot be diagnosed, and hence not treated intelligently. It can be diagnosed, and in the vast majority of instances the diagnosis is easy.

The symptoms of the patient give some assistance, but are often very misleading, and the diagnosis is never settled, except in advanced malignancy, till a test-meal is given, and this test-meal, at the proper time, drawn and analyzed chemically. This shows just what the stomach can do, and also shows its points of disease, and opens up the way for the proper treatment. This is an easy procedure, and gives a key to the situation that is obtainable in no other way.

Many cases bring big surprise, and to exemplify the fact that it is easy to be misled by symptoms I will briefly cite a couple of cases now under treatment: Doctor G. presented all the symptoms of a chronic mucous gastritis, with no symptoms of hyperacidity at all, and he was of the opinion that he had anacidity. Analysis of his test-meal showed pronounced hyperacidity, and treatment for same is bringing about steady improvement in his case.

A second case, Mr. G., presented many of the symptoms of chronic acid gastritis, and I would have made the diagnosis accordingly had not the test-meal analysis shown me that his free Hcl was less than half up to the normal standard. It takes only a casual glance to see that treatment of either of these two cases from their symptoms alone would have resulted in making them worse instead of better.

---

### VERATRUM VIRIDE IN MANIA.

Any physician who has not employed veratrum viride in acute mania has missed the best agency which is available for the cure of these distressing cases. It is one of the greatest advantages a physician can have to see the feverish sufferer, under the application of this remedy, pass from absolute sleeplessness into a state of quiet rest. That many cases which would otherwise go on to death are saved by the use of this remedy is a fact beyond question. The fear which many practitioners have of using veratrum viride, on account of the varying strength of its various preparations, must, of course, be met when the drug is employed, by the use of Norwood's tincture.—*American Medical Journal.*

